# Left ventricular hypertrabeculation/noncompaction: a cardiac manifestation of myopathy?

**DOI:** 10.1590/S1516-31802007000100013

**Published:** 2007-01-04

**Authors:** Josef Finsterer, Claudia Stöllberger, Georg Blazek

We read with interest the review article by Moreira et al. on left ventricular hypertrabeculation/noncompaction (LVHT).^1^ However, we want to add some points and raise some concerns. It is well known that LVHT is frequently associated with neuro-muscular disorders, like dystrophinopathy, dystrobrevinopathy, myotonic dystrophy, zaspopathy, myoadenylate-deaminase deficiency, Charcot-Marie-Tooth disease, mitochondrial disorder, Barth syndrome, Friedreich's ataxia, or Pompe's disease.^2^ Thus, LVHT is genetically heterogeneous and may not only be associated with mutations in the G4.5 (taffazin), dystrobrevin, or dystrophin genes, but also with mutations in the lamin A/C, cypher/ZASP, GAA, DMPK, AMPD1, LDB3, mitochondrial, frataxin, or PMP22 genes. Additio nally, LVHT has been described in single patients with rare genetic non-neuromuscular syndromes, like Turner syndrome, Ohtahara syndrome, Roifman syndrome, Noonan syndrome, nail patella syndrome, Melnick-Needles syndrome, MIDAS (MLS) syndrome, DiGeorge syndrome (22q11 deletion), congenital adrenal hyperplasia, distal 4q trisomy and distal 1q monosomy, monosomy 1p36, distal chromosome 5q deletion, trisomy 11, or trisomy 13.^2^

Furthermore, it is important to mention that LVHT is not exclusively a congenital disorder, but may rarely occur during the lifetime of individual patients.^3^ The exact pathogenesis of acquired LVHT, however, is unknown, but several pathogenetic scenarios for its development have been outlined, such as: 1) compensation for a myocardium that is impeded from ejecting physiological stroke volumes due to reduced contractility, by enlargement of the left ventricular surface, particularly of the segments most demanded during systole; 2) dissection of the endocardium and myocardium due to insufficiently functioning gap junctions; 3) penetration of persisting sinusoids into the left ventricular cavity and transformation into trabeculations; 4) frustrated attempts at hypertrophying the insufficiently contracting myocardium; 5) enlargement of the endocardial layer for more efficient oxygenation via the endocardium; or 6) attempts by the impaired myocardium to resist against an impeding dilatation by tightening the myocardial structure.^2^

Though repeatedly reported in single cases, it is debatable whether LVHT is associated with an increased rate of stroke or embolism due to thromboembolism from the deep inter-trabecular recesses.^4^ In a study on 62 of our own patients with LVHT, we did not find that these patients have an increased risk of developing stroke or embolism.^5^ A further argument against an increased risk of embolic events in LVHT patients is the existence of animals in which LVHT is a physiological condition without an increased rate of morbidity from thromboembolism. Thus, we regard it as not justified to prescribe oral anticoagulants generally to all LVHT patients. It is recommended only if there is documented atrial fibrillation or severe systolic dysfunction that administration of oral anticoagulants is indicated.

Overall, it has to be stressed that patients with LVHT carry an increased mortality risk and thus require thorough cardiological and neurological surveillance. Furthermore, patients with LVHT need to be investigated for possible neuromuscular disorders by a neurologist specialized in this topic. Anticoagulation is indicated only if there is simultaneous atrial fibrillation or severe left ventricular dysfunction. Since LVHT occurs in families, it is also important to investigate at least first-degree family members for LVHT.
