# Supplementary material for: Left ventricular hypertrabeculation/noncompaction: a cardiac manifestation of myopathy?
Source: Sao Paulo Med J. 2007 Jan 4;125(1):63–4. doi: 10.1590/S1516-31802007000100013 (PMC11014708; doi:10.1590/S1516-31802007000100013)
Supplement: Related texts

# Reply: RESPONSE

**Authors:** Fábio Cañellas Moreira, Marcelo Haertel Miglioransa

We consider ourselves honored to receive the comments from Dr. Finsterer et al. about our article. In our review,^1^ we had not identified any acquired left ventricular hypertrabeculation/noncompaction (LVHT) cases, and Dr. Finsterer's contribution regarding the instances in which such cases can occur is of great value.^2^ In fact, it is important to investigate neuromuscular disorders in patients for whom a diagnosis of apparent LVHT alone is proposed.

As Dr. Finsterer pointed out, recent prospective studies have shown that there is no increased risk of thromboembolism in patients with LVHT,^3,4^ and we agree that anticoagulation is indicated only if there is simultaneous atrial fibrillation or severe left ventricular dysfunction.^5^

Finally, we corroborate the importance of investigating at least the first-degree relatives of LVHT patients, in order to attempt to provide adequate genetic counseling for their families.

## References

[1] Moreira FC, Miglioransa MH, Mautone MP, Muller KR, Lucchese F (2006). Noncompaction of the left ventricle: a new cardiomyopathy is presented to the clinician. Sao Paulo Med J.

[2] Finsterer J, Stöllberger C, Schubert B (2004). Acquired left ventricular hypertrabeculation/noncompaction in mitochondriopathy. Cardiology.

[3] Sharma ND, McCullough PA, Philbin EF, Weaver WD (2000). Left ventricular thrombus and subsequent thromboembolism in patients with severe systolic dysfunction. Chest.

[4] Stöllberger C, Finsterer J (2005). Left ventricular hypertrabeculation/noncompaction and stroke or embolism. Cardiology.

[5] Lilje C, Razek V, Joyce JJ (2006). Complications of non-compaction of the left ventricular myocardium in a paediatric population: a prospective study. Eur Heart J..
